# Tricuspid Valve Infective Endocarditis With Biventricular Thrombi: An Unusual Presentation

**DOI:** 10.1155/cric/2016181

**Published:** 2026-09-08

**Authors:** Genevieve Phipps, Sarteg Singh, Shane Preston

**Affiliations:** ^1^ Department of Cardiology, Cairns Hospital, Cairns, Queensland, Australia, health.qld.gov.au; ^2^ Department of Respiratory Medicine, Gold Coast University Hospital, Gold Coast, Queensland, Australia, qld.gov.au

## Abstract

**Background:**

Biventricular thrombi (BVT) is a rare presentation associated with high morbidity and mortality due to the potential sequela of life‐threatening complications including embolic events and cardiac failure. As an uncommon entity, there is limited research on best practice for the management of BVT.

**Presentation:**

We present a case of BVT with *Streptococcus anginosus* and *Staphylococcus hominis* concomitant bloodstream infection, tricuspid valve infective endocarditis (TVIE), septic emboli and decompensated dilated cardiomyopathy (LVEF 20%–25%). Guideline directed heart failure management, antimicrobials and anticoagulation were initiated with a remarkable recovery within 2 months.

**Discussion:**

Given the rarity of this presentation, a multidisciplinary approach was taken to guide management. Follow‐up echocardiography after completion of antimicrobial therapy demonstrated an LVEF of 53%, no detectable thrombus and resolution of congestive symptoms. Although there is minimal population‐based evidence, this case supports a conservative approach for the treatment of TVIE, BVT and its potential sequelae in select patients.

## 1. Case Description

A 54‐year‐old male, active intravenous drug user (IVDU) with excess alcohol intake and a known history of dilated cardiomyopathy (DCM) presented to the emergency department with a 3‐day history of anasarca and a 3‐week history of fever, haemoptysis, dyspnoea and malaise following a recent toothache. He had otherwise been in good health with no recent travel, procedures or relevant exposures. Last use of methamphetamines was one week prior to presentation. At time of admission the patient was non‐compliant with his prescribed DCM management.

On presentation the patient was febrile (38.1°C), tachycardic (102 bpm), tachypnoeic (RR 24) and hypertensive (144/97). Physical findings included an elevated jugular venous pressure with observed V waves, reduced air entry and bibasal crackles, new systolic murmur, pulsatile liver and bilateral pitting oedema to the hip. There were no peripheral stigmata of infective endocarditis (IE). Initial laboratory findings included a mild neutrophilia with marked elevation in BNP and troponin. Blood cultures on day of admission grew *Streptococcus anginosus* and *Staphylococcus hominis* (Table 1).

**Table 1:** Pertinent blood results from day of admission and positive blood culture results from serial cultures.

**Bloods**	**Results**	**Normal range**	

Hb	143 g/L	135–180 g/L	
WCC (neutrophils)	12.4 (10.7) × 10^9^/L	4.0–11(2.00–8.00) × 10^9^/L	
CRP	121 mg/L,	< 5.0 mg/L	
Albumin	16 g/L	35–50 g/L	
Creatinine	121 mmol/L	60–110 mmol/L	
AST	90 U/L	< 35 U/L	
ALT	153 U/L	< 45 U/L	
ALP	148 U/L	30–110 U/L	
GGT	61 U/L	< 55 U/L	
BNP	2100 ng/L	< 100 ng/L	
TnI	520 ng/L	< 54 ng/L	

**Admission day**	**Speciation**	**Time**	**Bottle #**

Day 1: morning	*S. anginosus*	31 h	one of two bottles
Day 1: afternoon	S. *hominis*	20.5 h	one of two bottles
Day 3	S. *hominis*	22 h	one of two bottles

**Figure 1:**
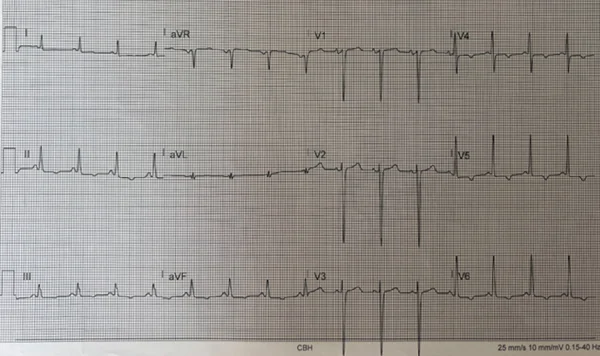
12‐lead ECG from day of admission.

**Figure 2:**
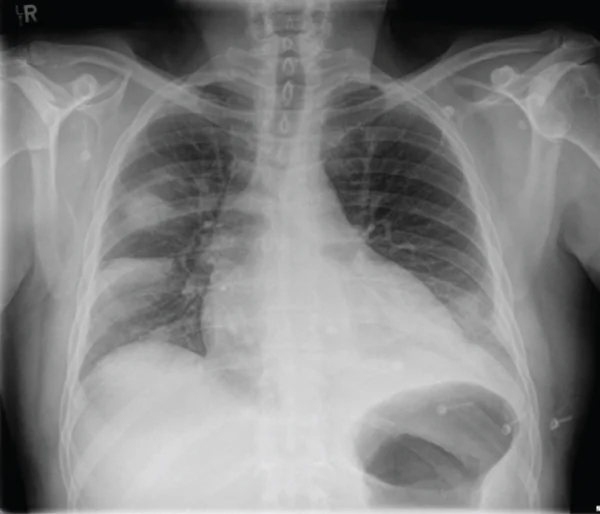
Postero‐anterior chest X‐ray on day of admission

**Figure 3:**
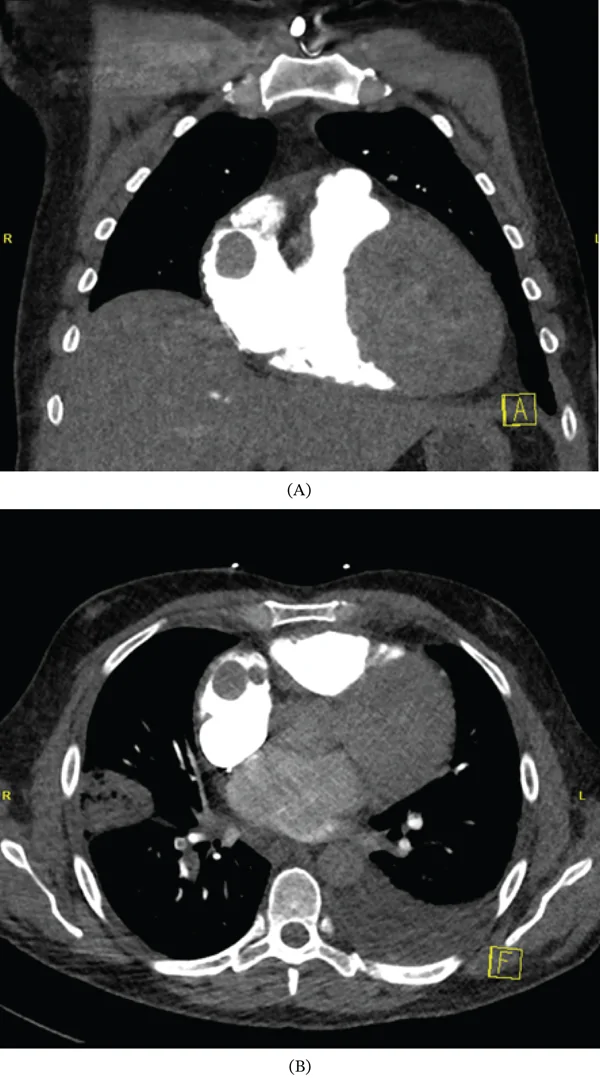
CT‐pulmonary angiogram on day of admission. (A) Coronal view. (B) Axial view.

**Figure 4:**
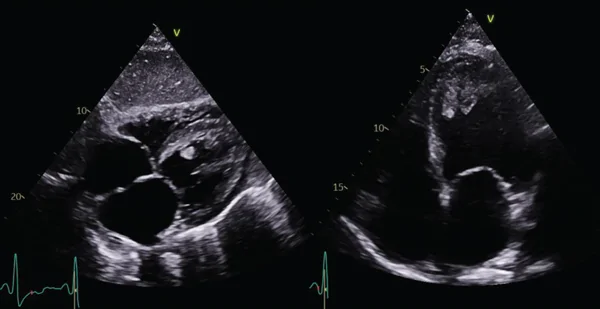
Four‐chamber view transthoracic echocardiogram on Day 2 of admission.

**Figure 5:**
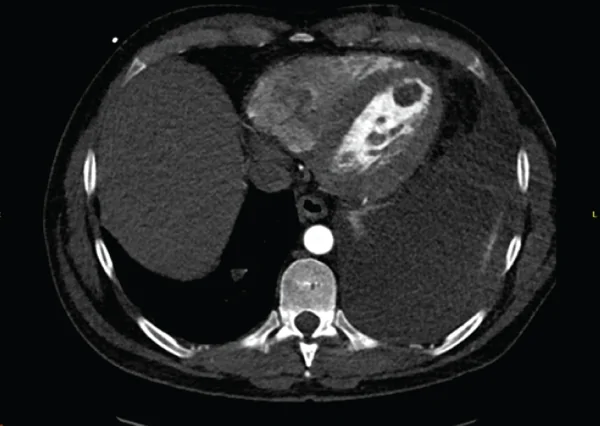
Axial slice of CT‐coronary angiogram on Day 9 of admission.

A 12‐lead ECG on admission showed sinus rhythm with new inferior T‐wave inversion (Figure 1). Previous ECG findings of LV hypertrophy were noted. Initial chest X‐ray was suggestive of multilobar pneumonia with extensive consolidation in the left lower lobe (Figure 2). A subsequent CT‐pulmonary angiogram (CTPA) identified extensive bilateral segmental pulmonary emboli with multifocal infiltrates (likely septic emboli) with early cavitation and a left parapneumonic effusion. A large filling defect was also observed in the anterior and superior aspect of the right atria and ventricle, reported as consistent with a mural or infected thrombus (Figure 3). Additional CT imaging did not identify evidence of septic emboli in the brain, abdomen or pelvis at time of admission. MRI brain later in admission identified focal lesions consistent with septic embolic events. On transthoracic echocardiogram (TTE) the following day, multiple large echogenic tethered masses were noted in both the RV and LV, with no apparent valvular involvement (Figure 4). Other abnormal TTE findings included severe global impairment of systolic function with a LVEF of 20%–25% and Grade III diastolic dysfunction. The LV was considered moderately dilated with mild concentric wall thickening. The left atrium was severely dilated, and the right atrium was moderately dilated. No significant valvular abnormalities were noted. CT‐coronary angiogram (CTCA) identified two foci of left‐sided interventricular thrombi with normal coronary arteries (Figure 5). On subsequent cardiac MRI (cMRI) on Day 9 of admission, RVEF and LVEF were severely reduced at 38% and 23% respectively, with a remaining LV apical thrombus of 20 mm in diameter and no late gadolinium enhancement.

Despite the lack of radiographic evidence of valvular involvement, the presentation was considered consistent with a clinical diagnosis of IE. Given the septic pulmonary emboli, the RV masses were presumed to be secondary to TVIE complicated by right ventricular thrombi. A periapical dental abscess was identified on an orthopantomagram X‐ray*. S. homini*s was again grown on repeat blood culture on Day 3 of admission. The origin of the left‐sided lesions remained contentious in the absence of tissue biopsy. The consensus amongst specialists from quaternary centres was that LV thrombi secondary to DCM were more probable than concurrent left‐sided endocarditis with septic vegetations. The patient was given the clinical diagnosis of *S. anginosus* and *S. hominis* concurrent bacteremia with native TVIE complicated by BVT, severe biventricular failure and septic embolisation to the lungs and brain.

The patient was not considered a suitable surgical candidate in view of lack of evidence supporting valvular involvement, the presumed embedding of the thrombus into the trabeculae and the patient′s significant comorbidities. Medical management included 6 weeks of iv cefazolin from the date of first negative blood culture, Warfarin therapy for anticoagulation and furosemide for diuresis. Transition from heparin infusion to warfarin therapy was delayed until Day 10 of admission, when repeat MRI brain confirmed there was no haemorrhagic transformation of the brain infarcts. For DCM management, bisoprolol, ramipril, spironolactone and dapagliflozin were also commenced. Repeat TTE with contrast 2 months postadmission following the completion of IV antibiotics showed an improved LVEF of 53% with no detectable thrombus or persisting vegetation. The patient reported no ongoing infective or CCF symptoms. Furosemide and spironolactone were ceased to reduce pill burden, and warfarin was ceased on subsequent follow‐up review.

## 2. Discussion

Right‐sided IE is an uncommon entity accounting for 5%–10% of all IE cases and is most often associated with IVDU [1]. *Staphylococcus aureus* is the most frequently implicated pathogen in right‐sided IE accounting for 60%–90% of cases. *Streptococcal* strains are also known to be associated with right‐sided IE, particularly in the setting of chronic alcoholism [1]. The patient in the presented case had multiple IE risk factors including alcohol abuse, active IVDU and DCM with medication non‐compliance. Although the patient had no radiographic evidence of valvular involvement, the clinical picture was considered consistent with IE and met Dukes‐ISCVID criteria for a possible diagnosis.

*S. anginosus* from the *milleri* family is a resident flora of the oral cavity and upper respiratory tract, associated with periodontal diseases including odontogenic abscesses [2]. In the presented case, the patient reported a toothache preceding onset of symptoms and orthopantomagram X‐ray confirmed presence of a dental abscess, potentially the seeding point of initial infection. Although a less common cause of bacteraemia, bloodstream infections with *S. anginosus* are commonly associated with abscess formation and are four times more likely than *Streptococcus pneumoniae* to be associated with IE [3]. Although rare, TVIE, septic embolisation and thrombus formation have been documented complications of *S. anginosus* bacteraemia [2]. However, on review of the available literature, there was no documented case of associated BVT.

*S. hominis*, a part of the normal skin flora and a commonly recognised contaminant of blood cultures, was cultured twice during admission. Although rare, there are reported cases of *S. hominis* native valve IE with embolic phenomena [4]. Given the low virulence properties of this bacterium and the concurrent finding of *S. anginosus* in the bloodstream, both positive blood cultures for *S. hominis* may reflect contamination. However, in the absence of biopsy, it is diagnostically challenging to attribute aetiology of the presented case to solely one isolate.

Isolated LV thrombus is not an entirely uncommon presentation in DCM [5]. However, BVT is considered highly atypical. The combination of high bacterial seeding with septic emboli from TVIE, underlying structural tissue damage secondary to DCM and ongoing IVDU likely all contributed to this atypical presentation. BVT is known to be associated with hypercoagulable inflammatory states including heparin‐induced thrombocytopenia, antiphospholipid syndrome, cardiomyopathies, myocarditis and myocardial infarctions [6]. Diagnosis of BVT is of high clinical importance as it carries significant risk of embolic event and cardiac failure due to reduction in EF [5]. Both complications were seen in the presented case and are associated with high morbidity and mortality. Histopathological examination remains the definitive method for establishing diagnosis of intracardiac masses. However, this is invasive and carries significant procedural risk. Consequently, a non‐invasive multimodality approach is more commonly favoured [7].

In this case, cardiac thrombi were incidentally visualised on the CTPA and confirmed on TTE, CTCA and CMRI. Diagnosis of intracardiac masses most commonly occurs through TTE, as it is low cost and widely accessible. Findings on TTE, such as significant pericardial effusion, right‐sided masses, heterogeneous echogenicity and invasion into surrounding tissue, are strongly suggestive of malignant origin [7, 8]. Contrast‐enhanced TTE can overcome limitations associated with poor acoustic windows, increasing diagnostic sensitivity for LV thrombus from 35% to 64%. Contrast‐enhancement can also assist in differentiating vascular tumours from thrombi [7, 9]. Similarly, the use of pulsed‐wave tissue Doppler imaging can assist in differentiating intracardiac masses from surrounding tissue and prognosticating embolic risk through measurement of mass peak antegrade velocity [10–11]. Conversely, transesophageal echocardiography (TEE) characteristically has poor visualisation of the left apex and is therefore not recommended in investigation for ventricular masses. However, TEE is considered the gold standard for detecting small mobile lesions (< 1 cm), such as vegetations or lesions involving the valve because of its superior temporal resolution compared with MRI [12–13].

Although all imaging modalities in the presented case identified the presence of thrombi, CMR is considered the gold standard for non‐invasive tissue characterisation of intracardiac masses with a reported sensitivity of 82%–88% and diagnostic accuracy of 92%–100%, although TEE remains superior for mobile valvular lesions [7, 12]. Signal intensity of T1‐ and T2‐weighted images assists in differentiating fatty tumours and melanomas, which demonstrate hyperintense T1 signal, from highly vascular tumours, malignant masses and cysts, which typically demonstrate hypointense T1 signal, with malignant masses often exhibiting hyperintensity on T2‐weighted images. Similarly, T1‐ and T2‐weighted signal characteristics can assist in thrombus tissue characterisation and determining thrombus age, with thrombi generally demonstrating progressively reduced T1 and T2 hyperintensity with increasing chronicity. Postcontrast tissue characterisation further assists in diagnosis; early gadolinium enhancement is suggestive of vascular tumours, whereas absence of enhancement is suggestive of thrombotic masses, and late enhancement is suggestive of malignant processes [7, 12]. Diagnostic accuracy of CMR requires gating, and image quality may be significantly reduced in the presence of tachyarrhythmias [14]. Unfortunately, CMR may also not be available in regional or rural environments, can be poorly tolerated in patients with claustrophobia, the prolonged imaging time may exclude haemodynamically unstable patients or those unable to lie flat for prolonged periods and is contraindicated in certain older non‐MR conditional cardiac devices [7]. Although the supporting evidence is less extensive than for CMR, cardiac CT is considered a suitable alternative in these patients, offering tissue characterisation and high spatial resolution beyond what is accessible through echocardiography alone. CT offers more rapid image acquisition, is generally better tolerated and facilitates surgical planning through definition of anatomical relationship between the cardiac mass and coronary arteries [7, 15]. Unfortunately, literature comparing imaging modalities is largely limited to LV thrombi/masses, likely due to the low case numbers of RV and BVT.

As a rare entity, there is limited data on best practice for the management of BVT. Literature on LV thrombus in DCM is supportive of initiation of anticoagulation for a minimum duration of 3–6 months with follow‐up imaging and potential ceasing of anticoagulation if LVEF improves to > 35% [16]. In a retrospective analysis, 62.3% of patients with LV thrombus secondary to DCM achieved thrombus regression over a median of 103 days with anticoagulation [17]. The monitoring of thrombus regression in the described case was complicated by variation in imaging modalities through the treatment timeline. However, repeat contrast‐enhanced TTE following completion of IV antibiotics revealed no detectable thrombus. Suggesting medical management of BVT with anticoagulation is a reasonable and effective clinical approach.

## 3. Conclusion

BVT is a rare presentation associated with a sequela of potentially life‐threatening complications. Clinicians should therefore have a high index of suspicion in atypical cardiac presentations with known hypercoagulable, pro‐inflammatory and structural risk factors for BVT. DCM is a well‐described risk factor for cardiac thrombi, although less commonly for BVT. Diagnosis without biopsy evidence can be complicated by low imaging sensitivity and may require multiple imaging modalities. Best practice for management remains elusive due to the rarity and heterogeneous nature of these presentations. However, case‐based findings and LV thrombus management guidelines are largely supportive of the use of anticoagulation as the mainstay of clinical management.

**Nomenclature**
BVT: biventricular thrombi
cMRI: cardiac magnetic resonance imaging
CTCA: computed tomography cardiac angiography
CTPA: computed tomography‐pulmonary angiogram
DCM: dilated cardiomyopathy
IVDU: intravenous drug user
LVEF: left ventricular ejection fraction
TEE: transesophageal echocardiography
TTE: transthoracic echocardiogram
TVIE: tricuspid valve infective endocarditis

## Funding

No funding was received for this manuscript.

## Disclosure

The authors have nothing to report.

## Consent

The patient in the presented report has provided informed consent. Written informed consent was obtained from the patient for publication of this case report and accompanying images.

## Conflicts of Interest

The authors declare no conflicts of interest.

## Data Availability

The data that support the findings of this study are available on request from the corresponding author. The data are not publicly available due to privacy or ethical restrictions.
